# Review of proximal splenic artery embolization in blunt abdominal trauma

**DOI:** 10.1186/s42155-019-0055-3

**Published:** 2019-03-18

**Authors:** Keith Bertram Quencer, Tyler Andrew Smith

**Affiliations:** Division of Interventional Radiology, University of Utah Department of Radiology, 30 N. 1900 E., Salt Lake City, UT 84132 USA

**Keywords:** Splenic embolization, Blunt abdominal trauma, Proximal splenic embolization, Splenic salvage, Splenic anatomy

## Abstract

The spleen is the most commonly injured organ in blunt abdominal trauma. Unstable patients undergo laparotomy and splenectomy. Stable patients with lower grade injuries are treated conservatively; those stable patients with moderate to severe splenic injuries (grade III-V) benefit from endovascular splenic artery embolization. Two widely used embolization approaches are proximal and distal splenic artery embolization. Proximal splenic artery embolization decreases the perfusion pressure in the spleen but allows for viability of the spleen to be maintained via collateral pathways. Distal embolization can be used in cases of focal injury. In this article we review relevant literature on splenic embolization indication, and technique, comparing and contrasting proximal and distal embolization. Additionally, we review relevant anatomy and discuss collateral perfusion pathways following proximal embolization. Finally, we review potential complications of splenic artery embolization.

## Background

The spleen has many important roles including T-cell proliferation and antibody production and phagocytosis of senescent red blood cells (Coccolini et al. 2017; Mebius and Kraal 2005). Therefore, in the setting of splenic trauma, splenectomy is avoided when possible. Avoiding splenectomy precludes the development of overwhelming post-splenectomy sepsis, a potentially fatal infection caused by encapsulated bacteria (Coccolini et al. 2017; Uranus and Pfeifer 2001; Lynch and Kapila 1996; Cullingford et al. 1991; Banerjee et al. 2013).

Trauma protocols are resource and institution dependent. In general, hemodynamically stable with significant blunt abdominal trauma are imaged with contrast enhanced computed tomography (CT). The abdomen is typically scanned in a portal venous phase (Dreizin and Munera 2012). American Association for the Surgery of Trauma (AAST) splenic laceration grade is based on CT angiography findings (Moore et al. 1994) (see Table 1).

**Table 1:** AAST splenic laceration grading by CT. Other findings on CT not included in AAST criteria but important for triaging patients include active extravasation and pseudoaneurysm

	Grade I	Grade II	Grade III	Grade IV	Grade V
Subcapsular hematoma extent	< 10% of surface area	10–50% of splenic surface area	> 50% of splenic surface area, ruptured subcapsular hematoma,	N/A	N/A
Laceration depth	< 1 cm	1-5 cm	>5 cm	N/A	N/A
Other				Laceration of vessels leading to > 25% devascularization	Shattered spleen, complete devascularization

Splenic preservation can be accomplished via three routes: 1-bedrest and close monitoring alone (typically for grade I or II); 2- endovascular splenic artery embolization combined with bedrest and close monitoring (AAST grade III-V splenic injuries or with CT scans demonstrating pseudoaneurysms, traumatic arteriovenous fistula or extravasation); 3-surgical repair (known as splenorrhaphy). Endovascular splenic embolization is effective; when splenic preservation is done without adjunctive splenic artery embolization, failure (defined as the need for subsequent splenectomy) is seen in approximately 40% of high grade injuries compared to 2% when embolization has been performed (Banerjee et al. 2013; Dreizin and Munera 2012; Moore et al. 1994; Requarth et al. 2011; Ahuja et al. 2015; Scatliff et al. 1975; Albrecht et al. 2002; McIntyre et al. 2005).

Endovascular splenic artery embolization can be performed distally or proximally depending on the injury pattern. Distal splenic artery embolization is preferred in cases of focal vascular injury (e.g. vessel truncation, pseudoaneurysm, focal extravasation) (Bessoud and Denys 2004). Distal embolization is often precluded given the predominantly multifocal injury pattern of blunt splenic injury (Scatliff et al. 1975). In cases of multifocal injury or when no focal angiographic abnormality is identified, but CT has demonstrated injury, proximal splenic artery embolization (PSAE) is performed (Imbrogno and Ray 2012).

PSAE works by decreasing the systolic arterial pressure in the spleen, promoting hemostasis and healing within the splenic parenchyma. Blood flow to the spleen is maintained via collaterals, which not only prevents infarction and abscess formation but also preserves splenic immune function (Imbrogno and Ray 2012; Bessoud and Denys 2004; Zmora et al. 2009). PSAE may also be performed outside the setting of trauma such as in cases of splenic artery aneurysm/pseudoaneurysm and in post liver transplant splenic artery steal syndrome (Loffroy et al. 2008; Saad 2012; Gu et al. 2012).

Splenorrhaphy can be used for splenic preservation involves suturing splenic defects and/or applying hemostatic agents to the areas of splenic injury. It is most helpful in cases of polytrauma where non-operative management is not possible or preferrable, and has been shown to be an effective method for splenic preservation (Tsaroucha et al. 2005). Splenic preservation is only a secondary goal in the management of trauma patients; unstable patients should undergo splenectomy. In patients with poly-abdominal trauma, treatment decisions are based on patients’ overall clinical picture.

In this review, we discuss the use of splenic artery embolization as part of non-operative management for splenic injuries caused by blunt abdominal trauma. We compare and contrast proximal and distal embolization. We review relevant anatomy focusing on where to perform proximal embolization and routes of collateral perfusion following proximal embolization. Additionally, we provide procedural tips and tricks and review potential complications from splenic artery embolization.

## Study selection

Relevant articles pertaining to splenic trauma, function, anatomy, and splenic embolization using PubMed (US National Library of Medicine, Bethesda, MD) were reviewed. No studies were excluded based on year of publication. Selected studies were evaluated and assigned a level of evidence grade based on adaptations from existing guidelines (Table 2). Search terms included splenic embolization, splenic trauma, blunt abdominal trauma, splenic laceration, splenic collateral circulation, spleen preserving distal pancreatectomy.

**Table 2:** Summary table of key studies on splenic embolization with a level of evidence designation. Levels of evidence are defined using the grading system adapted from the American Society of Plastic Surgeons and Johns Hopkins nursing evidence-based practice: Models and Guidelines (Burns et al. 2011; Dang and Dearholt 2017) - (see Table 5)

Title, Author Year	Number of Patients	Study Design	Key Point(s), Data, Summary	Level of Evidence
Splenic trauma: WSES classification and guidelines for adult and pediatric patients (Coccolini et al. 2017).	NA	Review	Surgical management guidelines for splenic trauma including AAST classification and recommendations for the use of non-operative treatments. Recommendations include: Consideration of angiography and/or embolization in stable patients with AAST grade I-III splenic injury. Angiography and embolization for stable AAST grade IV-V splenic injuries.	IV
Trauma center variation in splenic artery embolization and spleen salvage: a multicenter analysis (Banerjee et al. 2013).	1275	Multicenter Meta-analysis	Centers with high use of splenic artery embolization have higher spleen salvage rates and fewer nonoperative management failures.	I
Nonoperative management of adult blunt splenic injury with and without splenic artery embolotherapy: a meta-analysis (Requarth et al. 2011).	10,157	Meta-analysis	Summarizes outcomes for patients with splenic injuries with non operative management. They found a higher failure rates in patients managed with observation alone compared with splenic artery embolization. Splenic artery embolization patients also showed significantly higher splenic salvage rates in grade 4 and 5 splenic injuries.	I
Transcatheter arterial embolization of splenic artery aneurysms and pseudoaneurysms: short- and long-term results (Loffroy et al. 2008)	17	Retrospective	Compared outcomes of endovascular treatment of splenic artery aneurysms and pseudoaneurysms. They found no major complications, and concluded embolization of splenic artery aneurysms and pseudoaneurysms is a safe and effective method of splenic preservation.	II
The anatomy of the fundic branches of the stomach: preliminary results (Gregorczyk et al. 2008).	NA	Descriptive laboratory study	Provides an anatomic description of the arterial vascularisation of the gastric fundus in 15 human specimens.	V
Outcomes of Proximal Versus Distal Splenic Artery Embolization After Trauma: A Systematic Review and Meta-Analysis (Schnuriger et al. 2011).	479	Meta-Analysis	Analyzes 15 studies regarding the use of both proximal and distal embolization in patients with splenic trauma. Summary of outcomes and complications for proximal vs distal splenic embolization.	I
Evaluation of the Amplatzer vascular plug for proximal splenic artery embolization (Widlus et al. 2008).	14	Retrospective	In these preliminary studies, Amplatzer vascular plugs were used successfully for proximal splenic artery embolization without any major complications.	II
Delayed presentation of splenic artery pseudoaneurysms following blunt abdominal trauma (Nance and Nance 1995).	2	Case report	Two patients with delayed presentation of splenic artery pseudoaneurysm following blunt abdominal trauma. Both vascular injuries were diagnosed on a follow up CT scan, highlighting the need for follow up imaging in patients with blunt abdominal trauma.	V
The impacts of different embolization techniques on splenic artery embolization for blunt splenic injury: a systematic review and meta-analysis (Rong et al. 2017).	876	Meta-Analysis	Comparison of PSAE vs distal embolization, and PSAE vs PSAE + distal embolization. Reports rates of success and severe complication. Lowest complications with PSAE, highest with combined proximal and distal embolization.	I
Conservation of the spleen with distal pancreatectomy (Warshaw 1988)	NA	Clinical examples, expert opinion	Authors describe their experience with preservation splenic vascular collateral pathways via the short gastric and gastroepiploic vessels during a distal pancreatectomy.	V
Laparoscopic spleen-preserving distal pancreatectomy: splenic vessel preservation compared with the Warshaw technique (Jean-Philippe et al. 2013).	140	Retrospective	Discusses collateral arterial pathways for splenic circulation, which are essential to splenic preservation following proximal splenic artery embolization.	II
Proximal splenic artery embolization for blunt splenic injury: clinical, immunologic, and ultrasound-Doppler follow-up (Bessoud et al. 2007).	37	Retrospective	Proximal splenic artery embolization for the treatment of splenic injury in blunt abdominal trauma is safe and preserves long term splenic function.	II
Splenic embolization revisited: a multicenter review (Haan et al. 2004).	140	Retrospective	Rebleeding following splenic embolization can occur in up to 24% of patients, but this is often treated successfully with re-embolization. Distal embolization often causes small splenic infarcts.	II
Non-operative management of blunt splenic injury: a 5-year experience (Haan et al. 2005).	109	Retrospective	Single center study showing hemodynamically stable patients with grade III – V splenic lacerations treated with PSAE have a higher likelihood of splenic salvage compared with those treated with observation alone.	II

## Splenic embolization-procedure

### Relevant anatomy

**Fig. 1:**
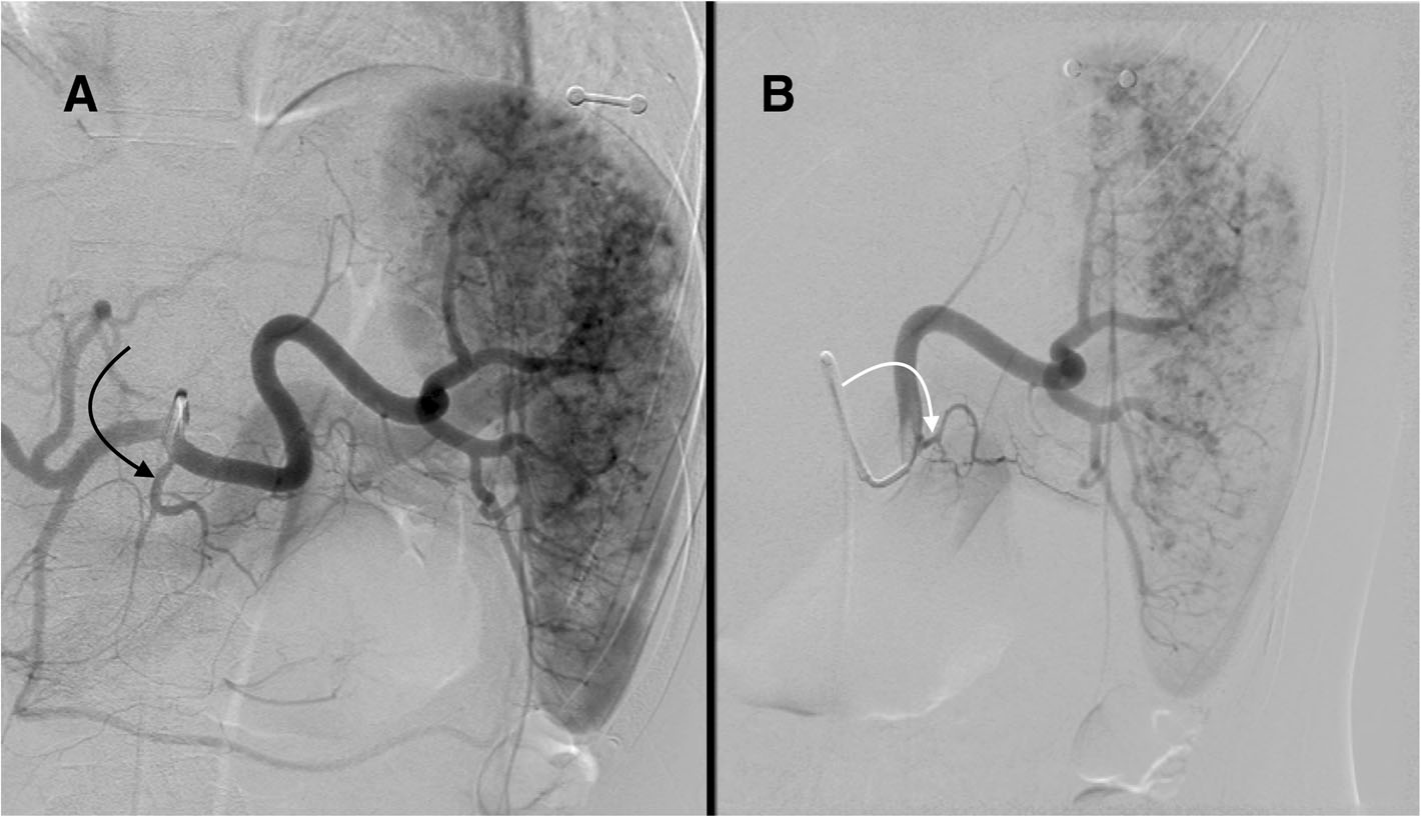
Celiac angiogram (**a**) in a 22 yo female status post rollover motor vehicle accident with grade III splenic laceration shows the dorsal pancreatic artery (curved black arrow) arising from the proximal splenic artery. The dorsal pancreatic artery arises from the proximal splenic artery in approximately 50% of cases. Selective splenic angiogram (**b**) shows the great pancreatic artery (curved white arrow) arising from the mid splenic artery. Note the multifocal areas of contrast pooling within the splenic parenchyma consistent with multifocal traumatic injury

**Fig. 2:**
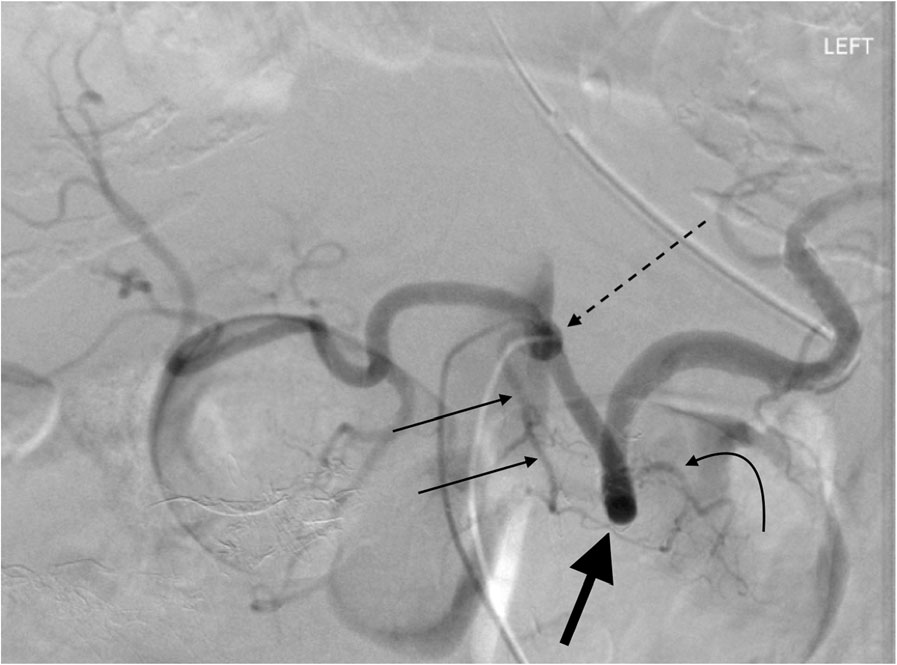
65 year old female undergoing celiac angiogram for upper gastrointestinal bleed. Celiac DSA showing the dorsal pancreatic artery (thin arrows) arising directly from the celiac trunk (dotted black arrow), which occurs in ~ 15% of cases. The great pancreatic artery (curved black arrow) arises from the mid portion of the splenic artery. Ideal placement of coils/plugs in proximal splenic artery embolization is between these two vessels. Transverse pancreatic artery (thick black arrow)

**Fig. 3:**
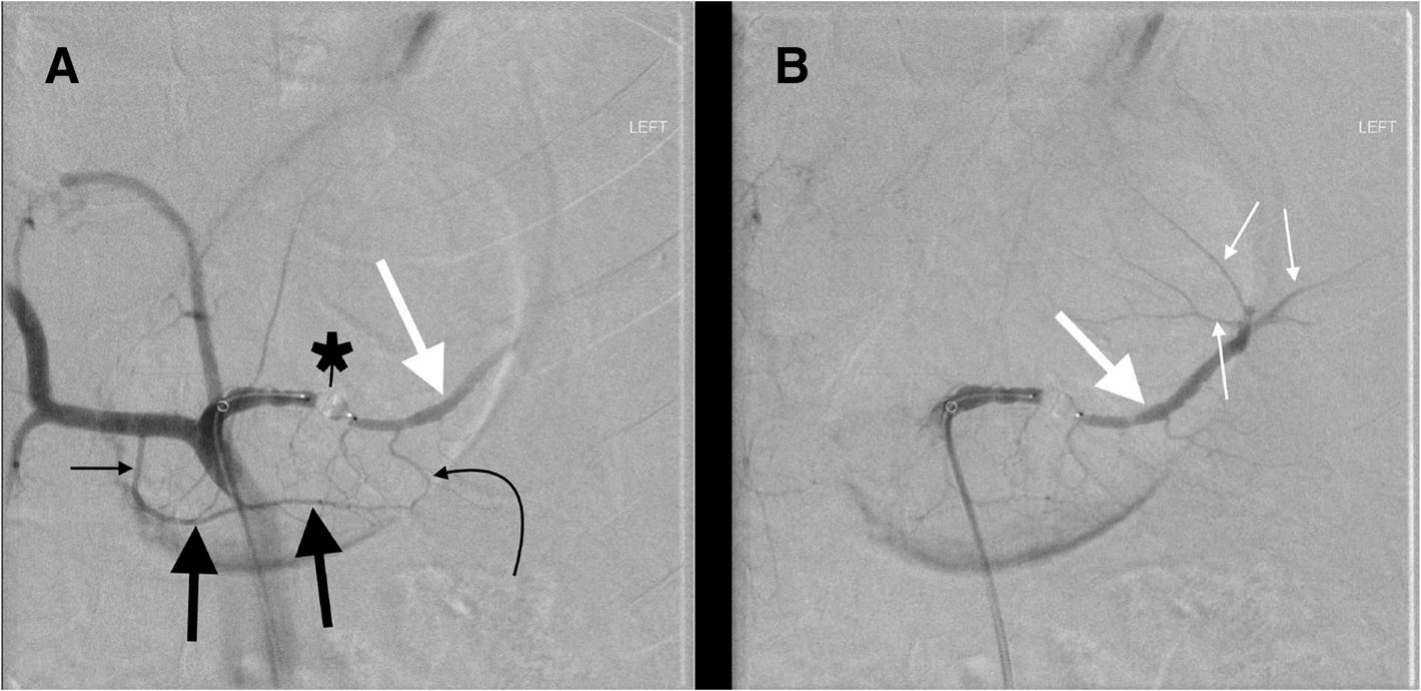
32 year old male in a snowmobile verses truck accident. **a** Celiac DSA after proximal splenic artery embolization with an AMPLATZER™ Plug (black star). Note the dorsal pancreatic artery (thin straight black arrow) originates from the common hepatic artery, a variation that occurs in approximately 20% of cases. Blood from the dorsal pancreatic artery then travels left along the transverse pancreatic artery (thick black arrows). Blood then flows retrograde up the great pancreatic artery (curved black arrow) reconstituting the mid/distal splenic artery (thick white arrow). **b** Subsequent image shows reconstituted flow in the mid/distal splenic artery (thick white arrow) with opacification of splenic artery branches (thin white arrows)

**Fig. 4:**
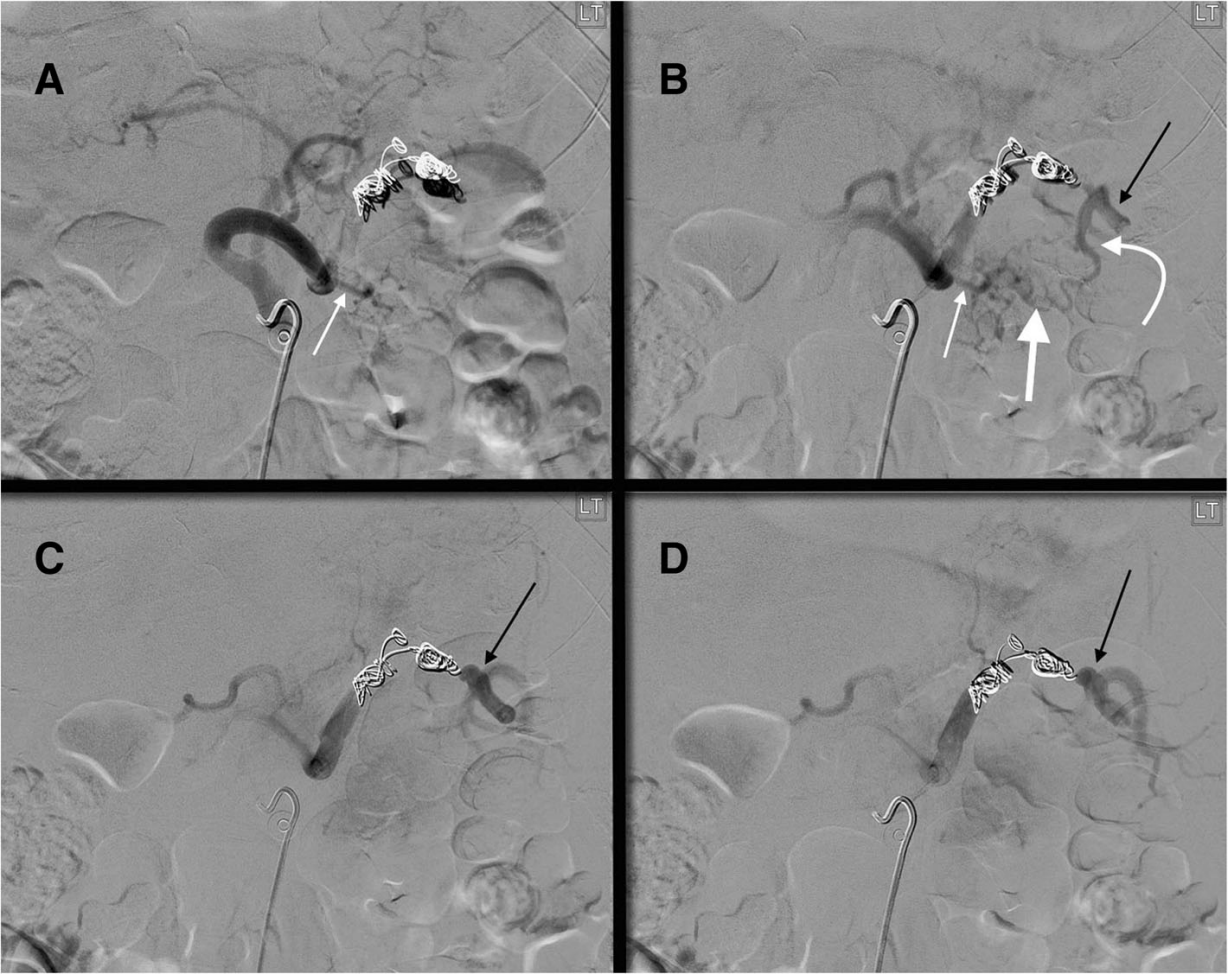
52 yo male status post motor vehicle accident with grade 3 splenic injury. After coils were mistakenly placed distal to the origin of the great pancreatic artery (thin white arrow **a**, **b**), splenic artery DSA shows there is reconstitution of the distal splenic artery (thin black arrows **b**-**d**) via a great pancreatic artery to transverse pancreatic artery (thick white arrow **b**) to caudal pancreatic artery (curved white arrow **b**) pathway. The caudal pancreatic artery arises from the distal third of the splenic artery (70% of cases) or an inferior polar branch of the splenic artery (30% of cases)

Along with the left gastric and common hepatic arteries, the splenic artery is one of three branches of the celiac trunk. The splenic artery supplies not only the spleen but also the body and tail of the pancreas and portions of the stomach. The first large branch of the splenic artery is typically the dorsal pancreatic artery, also known as the posterior pancreatic artery. This vessel most commonly arises from the proximal splenic artery (40–51% of cases; Fig. 1), but may also arise from the celiac trunk (3–28% of cases; Fig. 2), common hepatic artery (17–22% of cases; Fig. 3) or superior mesenteric artery (15–46% of cases) (Baranski et al. 2016; Bertelli et al. 1998; Okahara et al. 2010). The dorsal pancreatic artery bifurcates into left and right branches; the left branch continues as the transverse pancreatic artery, which runs parallel to the splenic artery. The second large branch of the splenic artery is the great pancreatic artery, which is also referred to as the arteria pancreatica magna and the greater pancreatic artery. This vessel typically arises from the mid portion of the splenic artery (see Figs. 1, 2, and 3). The caudal pancreatic artery is the most distal pancreatic branch, arising from the distal splenic artery in approximately 70% of cases or the inferior polar artery in the remaining 30% (Macchi et al. 2014) (Fig. 4). When performnig PSAE, the ideal placement of plugs/coils is between the dorsal pancreatic artery and great pancreatic artery.

### Embolization technique

Splenic artery embolization is typically performed via a trans-femoral approach. The celiac trunk is engaged using a 5 Fr reverse curve catheter, such as a Cobra, Sos or Simmons catheter. Celiac angiogram using digital subtraction angiography is then done, with a flow rate of 5–7 mL/second for a total volume of 20–28 mL. Images are evalutated for splenic artery patency, tortuosity, size, and pattern of injury. Common angiographic findings of splenic injury in blunt abdominal splenic trauma include arteriovenous fistula (direct connections between arteries and veins), pseudoaneurysm (focal outpouching of a vessel), vasospasum/truncation (abrubt vessel cutoff) (Madoff et al. 2005). Frank extravasation of contrast (extravascular contrast which diffuses from site of injury) can be seen, but is rare (Madoff et al. 2005). The origin, patency and course of other branches of the celiac trunk such as the left gastric artery, the gastroduodenal artery and the right gastroepiploic artery are evaluated as these branches are important in supplying collateral perfusion to the spleen. A catheter is then advanced selectively into the splenic artery and angiogram is then done with further evaluation of splenic artery diameter (typically between 5 and 9 mm in diameter) and splenic parenchymal injury. The origins of pancreatic branches such as the dorsal pancreatic artery and the great pancreatic artery are noted. Typical flow rates for selective splenic artery angiogram are 5–6 mL/second for a total volume of 15–18 mL.

### Proximal versus distal splenic artery embolization

PSAE is performed in cases of multifocal injury, whereas distal embolization is reserved for cases of focal vascular injury (Table 3). There is no significant difference in efficacy of splenic salvage between proximal and distal embolization (Schnuriger et al. 2011). There is, however, a higher rate of small splenic infarctions with distal splenic embolization (Imbrogno and Ray 2012; Schnuriger et al. 2011; Killeen et al. 2001). Another advantage of PSAE over distal embolization is faster procedure times, which is important in trauma patients whose hemodynamic stability can change quickly. A quicker procedure also allows for timely treatment of other injuries, and it helps reduce radiation dose (Imbrogno and Ray 2012). A theoretical disadvantage of PSAE is rebleeding distal to the coils/plugs; endovascular re-intervention would necessitate navigation through collaterals to perform subsequent distal embolization (Imbrogno and Ray 2012). This has not occurred in any published series to date. In cases where splenic injury is present but the angiography is negative, PSAE is preferred (Imbrogno and Ray 2012).

**Table 3:** Proximal vs Distal Splenic Embolization in Trauma Patients (Ahuja et al. 2015; Imbrogno and Ray 2012; Schnuriger et al. 2011; Killeen et al. 2001)

Type	When to Perform	Goal of Therapy	Advantages	Disadvantages	Embolics	Potential Complications
Proximal	Multifocal injury CT laceration without angiographic correlate	Decrease spleen parenchemyal perfusion pressure.	Shorter procedure times, lower radiation dose	Inability to easily perform future embolization.	Coils and/or plugs	Coil migration^a^
Distal	Trauma patients with focal vascular injury	Selection of specific injured vessels	Selective embolization	Increased procedure time, greater risk of infarction	Coils and/or particles	Splenic infarct and/or abscess formation

^a^Occurs in < 1% of cases (Haan et al. 2004)

### Distal embolization

To perform distal embolization, a microcatheter and microwire are advanced to the site of the vessel injury causing the extravasation, pseudoaneurysm or arterio-venous fistula. Once in proper position, embolization with particles, glue (such as N-butyl cyanoacrylate), gelfoam and/or coils is performed. Embolization using glue is an effective technique that can be used in patients with coagulopathy, however due to rapid polymerization of glue material, there is a small risk of embolization too proximal to the desired area of treatment (Loffroy et al. 2008). Completion selective and then proximal splenic angiograms are done after distal embolization to ensure desired occlusion of the injured vessel.

### Proximal embolization

For PSAE, vascular plugs and/or coils can be used (Table 4). The desired site of embolization for PSAE is between the dorsal pancreatic artery and great pancreatic artery (Widlus et al. 2008; Sclafani et al. 1991). Some operators prefer 0.035″ coils given their greater radial strength and wall apposition, which minimizes the risk of distal migration in the high flow splenic artery. Coils should be sized to be 20–30% larger and plugs should be sized 30–50% larger than the target vessel (Vaidya et al. 2008). Some operators prefer using vascular plugs for PSAE as they can be precisely placed (Norotsky et al. 1995). The AMPLATZER™ Family of Vascular Plugs (AVP; Abbott Laboratories, Chicago, IL) is the only plug large enough for the proximal splenic artery embolization. These plugs come in three varieties, the AVP, the AVP II and the AVP-4 (Vaidya et al. 2008). The AVP-4, which can be delivered through any catheter that accepts a 0.038″ wire, is infrequently used because the largest diameter they come in is 8 mm (maximum target vessel diameter of  5.5 mm). The AVP II comes in a wider variety of diameters. The 10 and 12 mm AVP II can be delivered through a 5 Fr sheath or a 6 Fr-guiding catheter.

**Table 4:** Pearls and Pitfalls of Splenic Artery Embolization

Plugs vs Coils	When performing PSAE, plugs may be to be technically easier and more precise than coils in the high-flow splenic artery can be technically challenging.
Coils	When using coils, strong consideration should be given to use of detachable coils.
	If coils are used 0.035″ coils are preferred to 0.018″ coils given their higher radial strength.
Plugs	To place. An AVP-II, the appropriately sized sheath is advanced just beyond the dorsal pancreatic artery. The plug is then advanced through the sheath, and the sheath retracted, uncovering the plug.
	Once proper position of the plug has been determined, the plastic vise is attached to the delivery wire and turned counterclockwise until the plug is detatched.

### Combined proximal and distal embolization

After distal embolization, PSAE can be performed. The decision on whether to perform PSAE after distal embolization depends on injury pattern, patient condition, local practice patterns and operator preference. The rationale for performing PSAE after successful distal embolization is that some vascular injuries may not be visible on initial angiogram and may lead to delayed bleed once the vasospasm subsides (Nance and Nance 1995; Campbell et al. 1991; Norotsky et al. 1995). This combined approach, however, leads to a higher rate of complications; a recent meta-analysis compairing serious complication rates (defined as those requiring further intervention, organ dysfunction, need for ICU management, or death) showed a more than doubled rate of complications in combined embolization (58.8%) compared to PSAE (18.2%) or distal embolization (28.7%) alone (Rong et al. 2017).

### Collateral routes of perfusion after PSAE

The left gastroepiploic artery arises from either the distal splenic artery or an inferior polar branch of the splenic artery. It runs along the greater curvature of the stomach and, in ~ 90% of cases, anastomoses directly with the right gastroepiploic artery, a terminal branch of the gastroduodenal artery (Egorov et al. 2011). The short gastric arteries are a group of 2–10 small terminal arteries arising from the distal splenic artery and its terminal branches (Egorov et al. 2011). They course within the gastrosplenic ligament where they anastomose with branches of the left gastric artery (Gregorczyk et al. 2008).

**Fig. 5:**
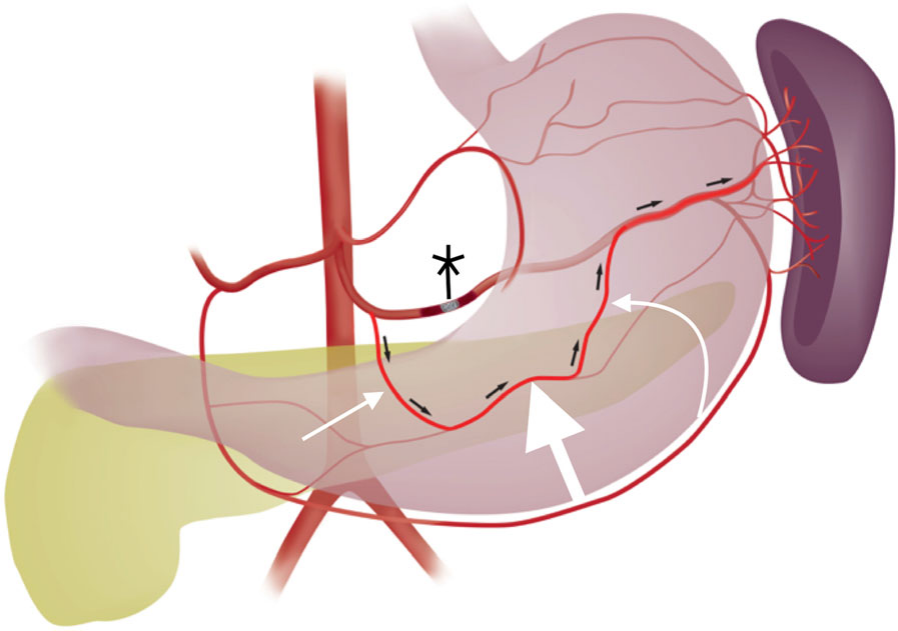
Schematic for the dorsal pancreatic (thin white arrow) to transverse pancreatic (thick white arrow) to great pancreatic artery (curved white arrow). Short black arrows denote the direction of flow. (Black star-coils in proximal splenic artery)

**Fig. 6:**
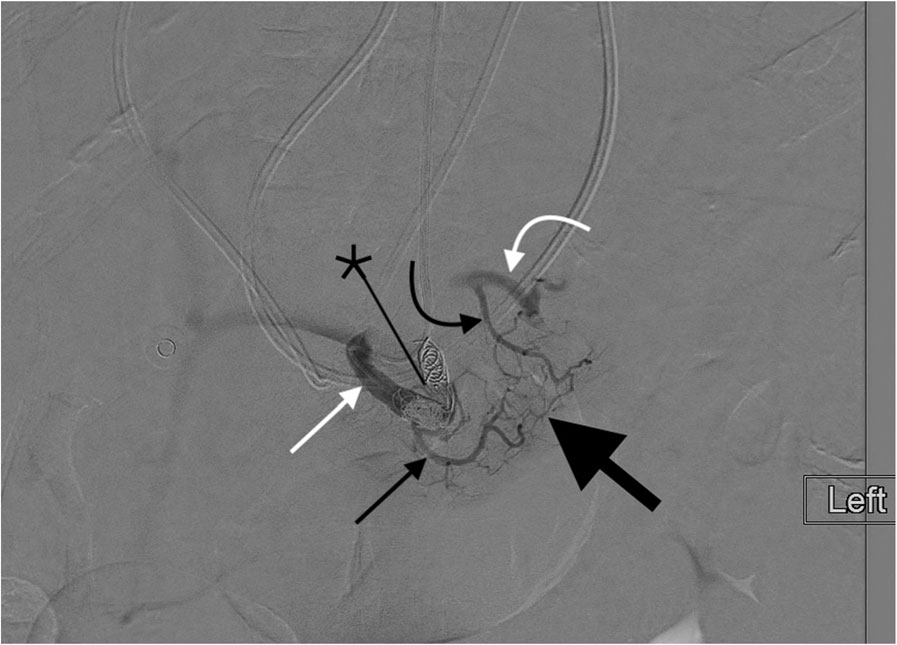
Transradial splenic angiogram following splenic trauma. After proximal splenic artery embolization using coils (black star), flow from the proximal splenic artery (straight white arrow) to the distal splenic artery (curved white arrow) is maintained via dorsal pancreatic (straight thin black arrow) to transverse pancreatic (straight thick black arrow) to great pancreatic artery (curved black arrow) pathway

**Fig. 7:**
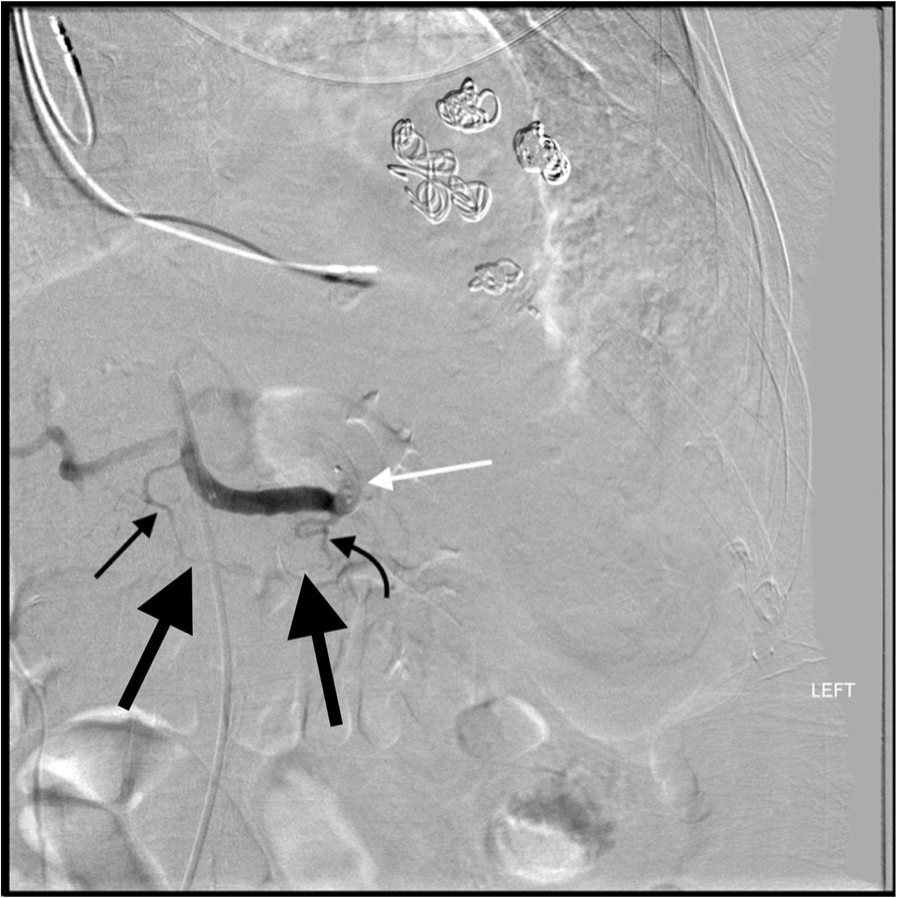
85 year old male with pancreatic cancer status post iatrogenic trauma following drain placement for a perisplenic abscess. Splenic artery DSA after embolization shows that the AMPLATZER™ Plug (white arrow) has mistakenly been placed distal to the great pancreatic artery (curved black arrow). This excludes collateral perfusion of the spleen via the dorsal pancreatic artery (straight thin black arrow) to transverse pancreatic (thick black arrows) to great pancreatic artery pathway

**Fig. 8:**
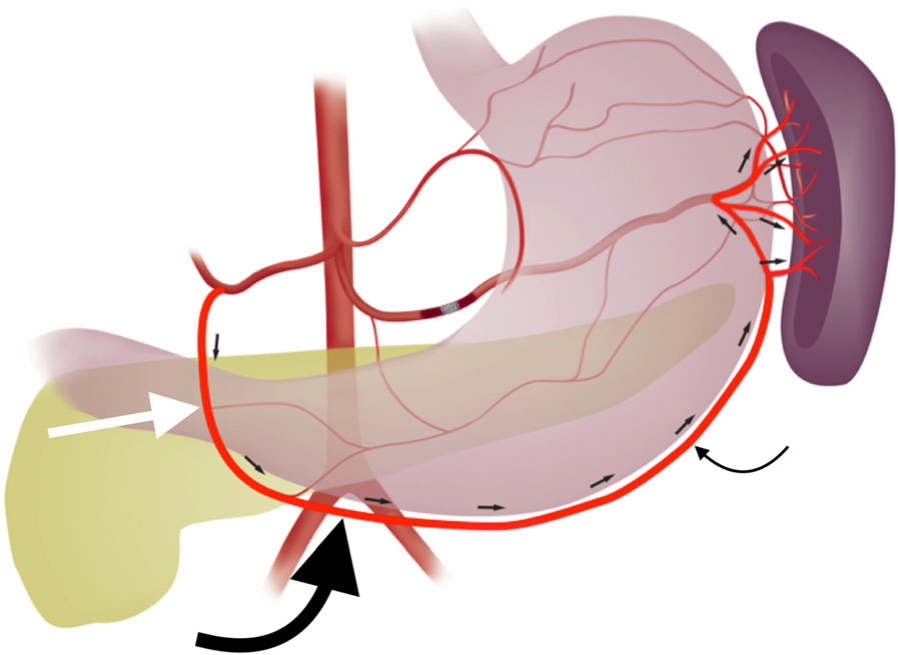
Schematic representation of collateral pathway of right gastroepiploic (thick curved black arrow) to left gastroepiploic artery (thin curved black arrow). The right gastroepiploic artery is a terminal branch of the gastrodoudenal artery (GDA; straight white arrow). It courses within the greater omentum along the greater curvature of the stomach. The left gastroepiploic artery may arise from the distal splenic or an inferior polar artery

**Fig. 9:**
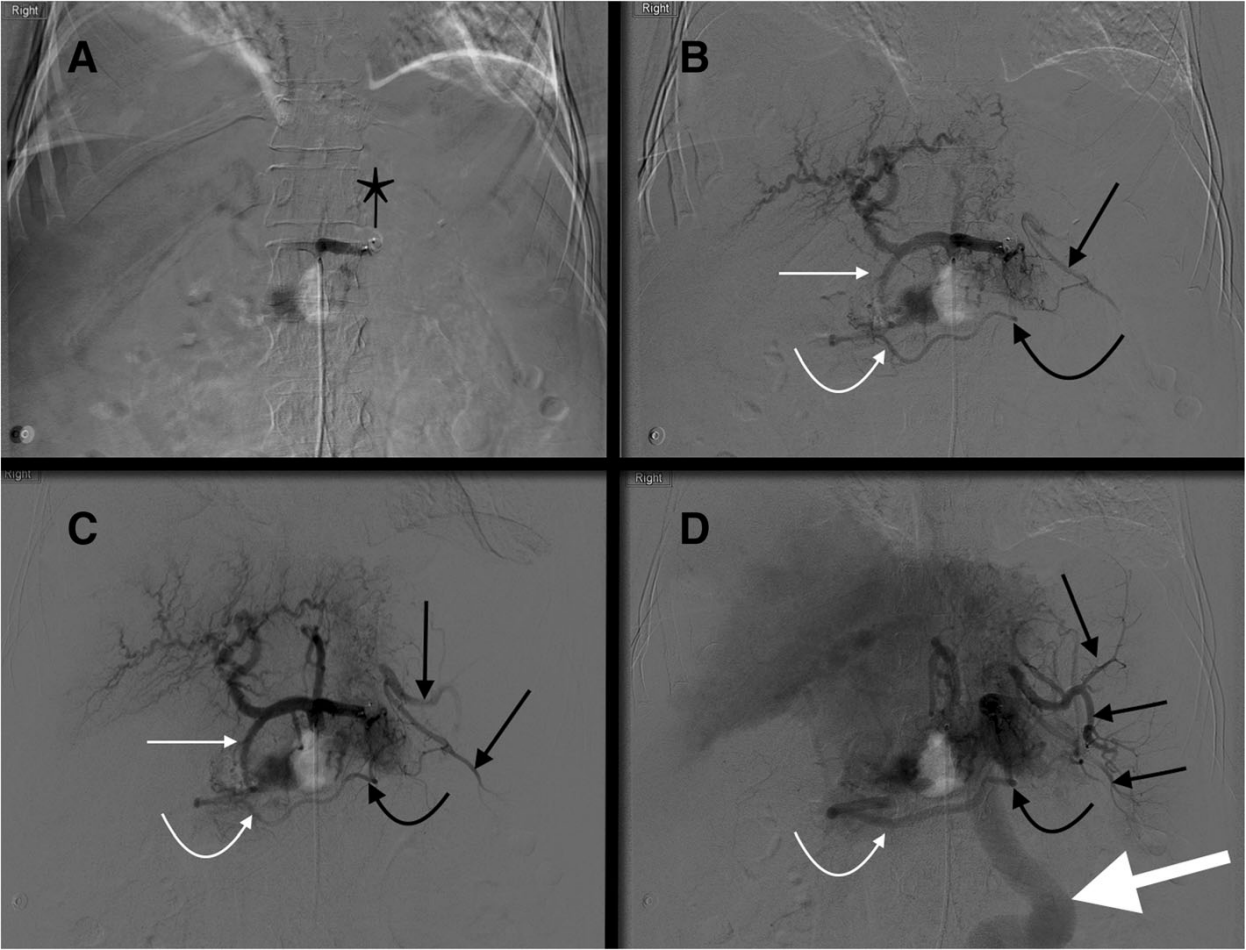
56 year old female, history of alcoholism, fall from standing height. Celiac artery angiogram after proximal splenic artery embolization with an AMPLATZERTM Plug (star **a**). This shows collateral perfusion to the spleen and distal splenic artery (straight black arrows **b**-**d**) via GDA (thin straight white arrow **b**, **c**) → right gastroepiploic (curved white arrow **b**-**d**) → left gastroepiploic pathway (curved black arrow **b**-**d**). Note that the parenchymal opacification of the spleen is markedly delayed compared to the liver, an expected finding after PSAE (**d**). Corkscrew intrahepatic arteries and the recannalized periumbilical vein with hepatopedal flow (thick white arrow **b**, **c**) are consistent with the patient’s known history of alcohol cirrhosis and portal hypertension

**Fig. 10:**
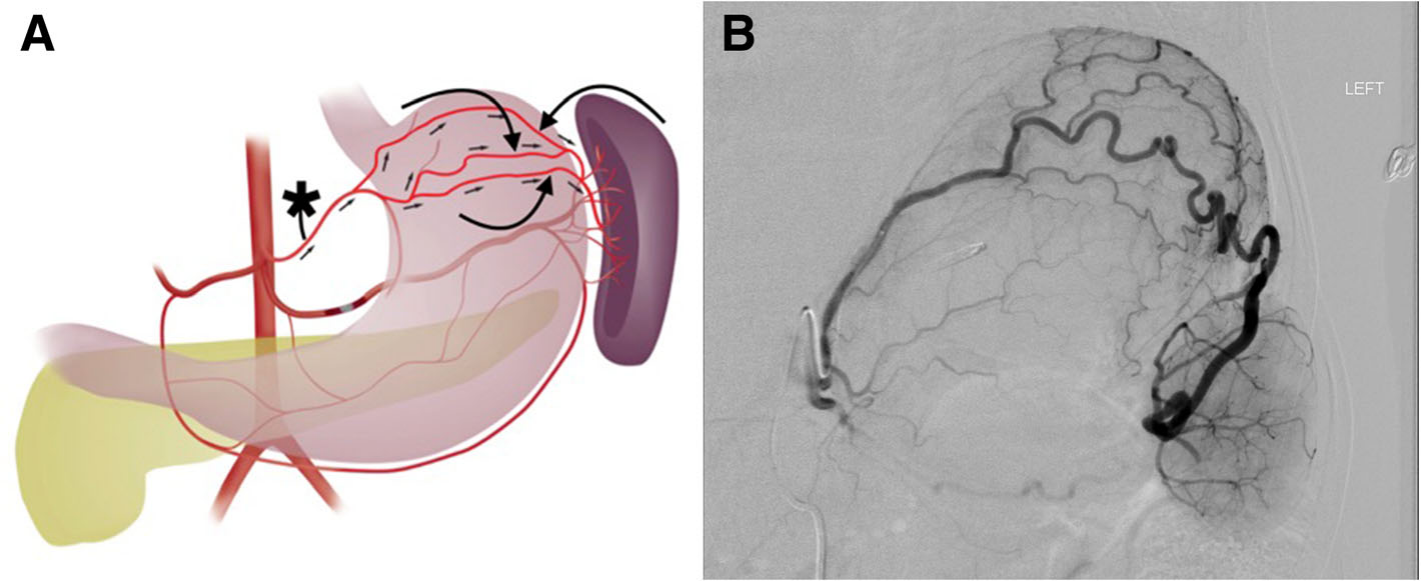
**a** Schematic of collateral splenic perfusion via the left gastric (star) to short gastric pathway (curved arrows). Note that the normal direction of flow is reversed in the short gastric arteries. **b** 39 year old female with history of splenic laceration caused by motor vehicle accident 11 years prior which was treated with a subtotal splenectomy as well as splenic artery and vein ligation. Left gastric artery angiogram done for bleeding gastric varices demonstrates the left gastric to short gastric to spleen collateral pathway

One collateral pathway to the spleen after PSAE is from the dorsal pancreatic artery to the transverse pancreatic artery to the great pancreatic artery which then feeds into the mid/distal splenic artery (Figs. 5 and 6). Care must be taken not to embolize distal to the great pancreatic artery; if embolization is performed distal to this artery, this route will longer be able to provide collateral flow to the spleen (Fig. 7). Other routes, such the great pancreatic artery to the caudal pancreatic artery may allow for collateral perfusion if embolization is performed distal to the great pancreatic artery (Fig. 4). Another route of collateral flow is from the right gastroepiploic artery to the left gastroepiploic, which then feeds into the distal splenic artery or an inferior polar branch (Figs. 8 and 9). The left gastric artery supplies an important collateral route after PSAE. The left gastric artery anastomoses with the short gastrics in the region of the fundus of the stomach, thereby supplying branches of the splenic artery (Fig. 10). These latter two pathways are relied upon to maintain splenic viability when distal pancreatectomy with splenic preservation and splenic vessel sacrifice (Warshaw’s technique) is performed (Warshaw 1988; Jean-Philippe et al. 2013). Finally, multiple collateral routes of collateral perfusion combining any of the above routes may also be seen.

## Post procedure

### Antibiotic therapy

Based on our literature review, there are no current antibiotic treatment guidelines for splenic artery embolization in the setting of trauma. At our institution, we routinely give 1 g of Cefazolin immediately prior to splenic artery embolization (Level V, see Table 5). Following PSAE, if the post embolization DSA shows good flow to the spleen we do not routinely use continue antibiotic therapy. If, however there is poor flow to the spleen on post procedure DSA, we give amoxicillin/clavulanic acid for 7 days to cover enteric flora. Following distal embolization, if a large territory was embolized we give 5 days of amoxicillin/clavulanic acid.

**Table 5:** Levels of Evidence Adapted from the American Society of Plastic Surgeons and Johns Hopkins nursing evidence-based practice: Models and Guidelines (Burns et al. 2011; Dang and Dearholt 2017)

Level	Description
I	High quality prospective RCTs, cohort studies with adequate power or systematic review of these studies
II	Lower quality prospective cohort, retrospective cohort study, RCT with untreated controls, or a systematic review/meta-analysis of these studies
III	Case-control study or systematic review/meta-analysis of these studies
IV	Case Series, consensus statements, society guidelines, practice guidelines
V	Expert Opinion: case report, clinical example, narrative reviews

### Splenic function after embolization

Splenic function is preserved after splenic embolization (Bessoud et al. 2007; Olthof et al. 2014). Preserved phagocytosis is confirmed by the absence of Howell-Jolly bodies (abdormal bsophilic nuclear remnants withing red blood cells due to deficient phagocytosis) after splenic embolization (Olthof et al. 2014; Pirasteh et al. 2012). Splenic dependent T-cell immunity is preserved after splenic embolization. The levels of two subpopulations of CD4+ T cells (CD4 + CD45RA+ and CD4 + CD45RO+), which are essential in antigen induced T-cell proliferation, are markedly diminished in asplenic patients but are normal after splenic embolization (Malhotra et al. 2010). Finally, antibody response to pneumococcal polysaccharide vaccine is preserved after splenic embolization but is blunted in asplenic patients (Olthof et al. 2014). Routine encapsulated organism antibiotics after splenic embolization is not needed.

### Complications

**Fig. 11:**
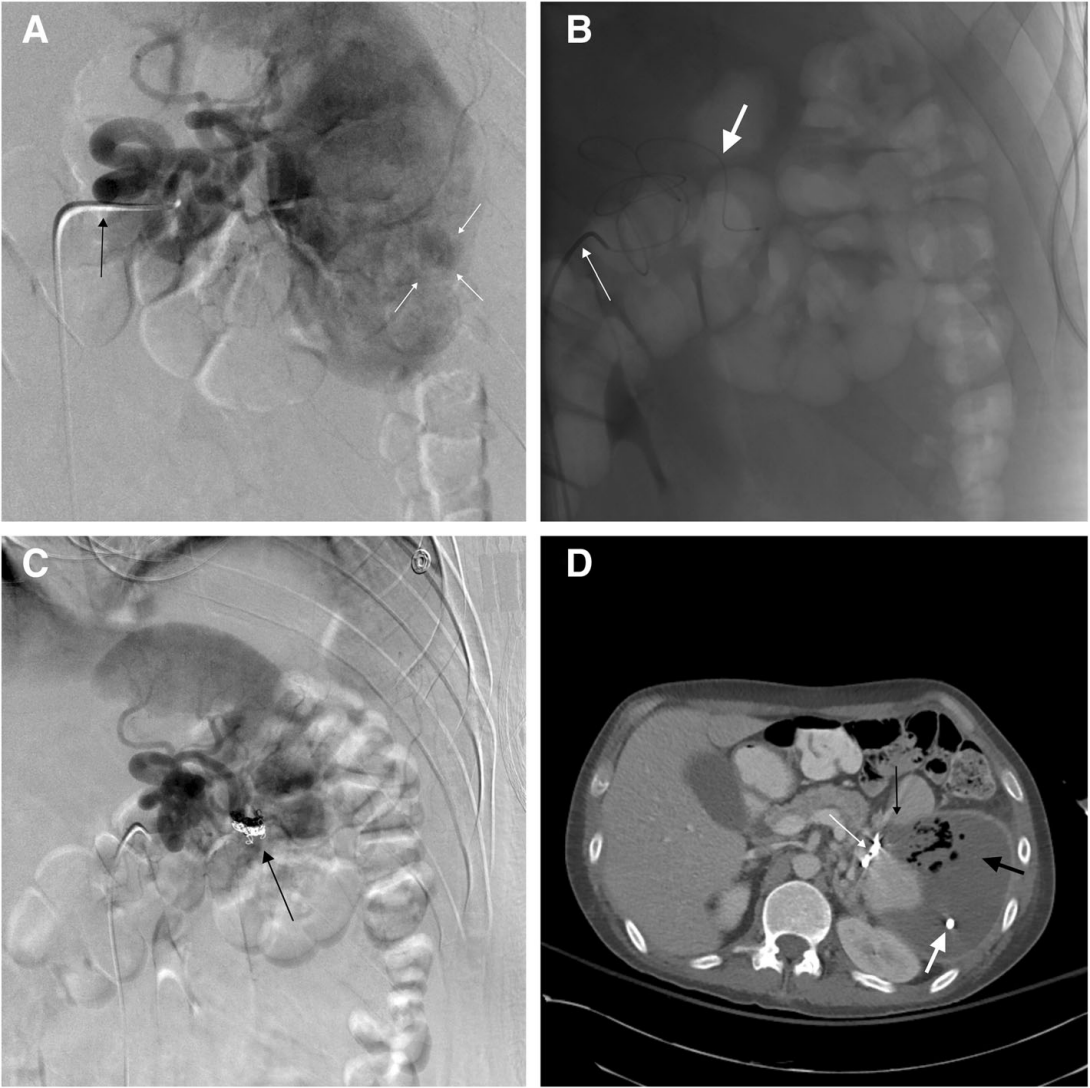
37 year old male with an AAST grade III splenic injury following high speed motor vehicle crash. **a** Splenic artery DSA with a catheter in the splenic artery (black arrow) demonstrating a focal pseudoaneurysm (white arrows). **b** A base catheter in the splenic artery (small white arrow), with a 5 F microcatheter fed through a tortuous splenic artery (large white arrow). The tortuosity and distance of this pseudoaneurysm precludes the use of traditional covered stents in this area. **c** Post coil embolization DSA showing a treated pseudoaneurysm. **d** One week after embolization, an axial contrast enhanced CT through the spleen demonstrates distal embolization coils (small white arrow), an area of focal infarction (small black arrow), and a splenic abscess (large black arrow). A partially visualized drain is present within the infected collection (large white arrow)

A multi-institutional trial assessing complication from splenic artery embolization found a major complication rate of 20%, and a minor complication rate of 23% (Ahuja et al. 2015; Haan et al. 2004). There is statistically no significant difference in the rate of major complications such as infection, infarction, or rebleeding requiring splenectomy when comparing proximal or distal splenic artery embolization (Schnuriger et al. 2011). The only complication discrepancy between PSAE and distal embolization is splenic infarct, with ~3x higher rate of infarct in distal embolizations (1.6% - 3.8% rate of major splenic infarctions in distal embolization; 0.0–0.5% for PSAE) (Schnuriger et al. 2011; Bessoud et al. 2007). Signs and symptoms of infarction include left upper quadrant pain and tenderness, fever, leukocytosis, nausea and vomiting, and an elevated serum lactate dehydrogenase (Lawrence et al. 2010). Splenic abscess (Fig. 11) often presents with persistent fever despite antibiotic coverage, left sided pleural effusion, and left upper quadrant pain (Lee et al. 2004).

## Conclusion

The spleen is commonly injured in blunt trauma. In hemodynamically stable patients, attempts are made at splenic preservation in order to sustain its immune function thereby preventing overwhelming post-splenectomy infection. PSAE plays an important role in non-operative management of splenic trauma. It works by decreasing perfusion pressure within the splenic parenchyma. At the same time, collateral flow keeps the splenic tissue viable, preserving its function and preventing infarction and abscess formation. Distal embolization can be performed in cases of focal vascular injury.

## Abbreviations

AAST: American Association for the Surgery of Trauma
APV: AMPLATZER™ Vascular Plug
CT: Computed tomography
DSA: Digital subtraction angiogram
PSA: Pseudoaneurysm
PSAE: Proximal splenic artery embolization
RTC: Randomized Controlled Trial

## References

[CR1] Ahuja C, Farsad K, Chadha M (2015). An overview of splenic embolization. Am J Roentgenol.

[CR2] Albrecht RM, Schermer CR, Morris A (2002). Nonoperative management of blunt splenic injuries: factors influencing success in age >55 years. Am Surg.

[CR3] Banerjee A, Duane TM, Wilson SP, Haney S, O'Neill PJ, Evans HL (2013). Trauma center variation in splenic artery embolization and spleen salvage: a multicenter analysis. J Trauma Acute Care Surg.

[CR4] Baranski AG, Lam HD, Braat AE, Schaapherder AF (2016). The dorsal pancreatic artery in pancreas procurement and transplantation: anatomical considerations and potential implications. Clin Transpl.

[CR5] Bertelli E, Di Gregorio F, Mosca S, Bastianini A (1998). The arterial blood supply of the pancreas: a review. V. The dorsal pancreatic artery. An anatomic review and a radiologic study. Surg Radiol Anat.

[CR6] Bessoud B, Denys A (2004). Main splenic artery embolization using coils in blunt splenic injuries: effects on the intrasplenic blood pressure. Eur Radiol.

[CR7] Bessoud B, Duchosal MA, Siegrist CA, Schlegel S, Doenz F, Calmes JM (2007). Proximal splenic artery embolization for blunt splenic injury: clinical, immunologic, and ultrasound-Doppler follow-up. J Trauma.

[CR8] Burns PB, Rohrich RJ, Chung KC (2011). The levels of evidence and their role in evidence-based medicine. Plast Reconstr Surg.

[CR9] Campbell D, Geraghty JG, McNicholas MM, Murphy JJ (1991). Delayed presentation of traumatic splenic arterio-venous fistula. Ir Med J.

[CR10] Coccolini F, Montori G, Catena F, Kluger Y, Biffl W, Moore EE (2017). Splenic trauma: WSES classification and guidelines for adult and pediatric patients. World J Emerg Surg.

[CR11] Cullingford GL, Watkins DN, Watts AD, Mallon DF (1991). Severe late post splenectomy infection. Br J Surg.

[CR12] Dang D, Dearholt S (2017). Johns Hopkins nursing evidence-based practice: model and guidelines.

[CR13] Dreizin D, Munera F (2012). Blunt polytrauma: evaluation with 64-section whole-body CT angiography. Radiographics.

[CR14] Egorov VI, Yashina NI, Zhurenkova TV, Petukhova MV, Starostina NS, Zarinskaya SA (2011). Spleen-preserving distal pancreatectomy with resection of the splenic vessels. Should one rely on the short gastric arteries?. JOP.

[CR15] Gregorczyk M, Dabkowska A, Tarka S, Ciszek B (2008). The anatomy of the fundic branches of the stomach: preliminary results. Folia Morphol (Warsz).

[CR16] Gu JJ, He XH, Li WT, Ji J, Peng WJ, Li GD (2012). Safety and efficacy of splenic artery coil embolization for hypersplenism in liver cirrhosis. Acta Radiol.

[CR17] Haan JM, Biffl W, Knudson MM (2004). Splenic embolization revisited: a multicenter review. J Trauma.

[CR18] Haan JM, Bochicchio GV, Kramer N (2005). Non-operative management of blunt splenic injury: a 5-year experience. J Trauma.

[CR19] Imbrogno BF, Ray CE (2012). Splenic artery embolization in blunt trauma. Semin Interv Radiol.

[CR20] Jean-Philippe A, Alexandre J, Christophe L, Denis C, Masson B, Fernandez-Cruz L (2013). Laparoscopic spleen-preserving distal pancreatectomy: splenic vessel preservation compared with the Warshaw technique. JAMA Surg.

[CR21] Killeen KL, Shanmuganathan K, Boyd-Kranis R, Scalea TM, Mirvis SE (2001). CT findings after embolization for blunt splenic trauma. J Vasc Interv Radiol.

[CR22] Lawrence YR, Pokroy R, Berlowitz D, Aharoni D, Hain D, Breuer GS (2010). Splenic infarction: an update on William Osler’s observations. Isr Med Assoc J.

[CR23] Lee CH, Leu HS, Hu TH, Liu JW (2004). Splenic abscess in southern Taiwan. J Microbiol Immunol Infect.

[CR24] Loffroy R, Guiu B, Cercueil JP, Lepage C, Cheynel N, Steinmetz E (2008). Transcatheter arterial embolization of splenic artery aneurysms and pseudoaneurysms: short- and long-term results. Ann Vasc Surg.

[CR25] Lynch AM, Kapila R (1996). Overwhelming post splenectomy infection. Infect Dis Clin N Am.

[CR26] Macchi V, Porzionato A, Picardi EE, Stecco C, Morra A, Bardini R (2014). Clinical anatomy of the caudal pancreatic arteries and their relevance in the surgery of the splenic trauma. Ital J Anat Embryol.

[CR27] Madoff DC, Denys A, Wallace MJ, Murthy R, Gupta S, Pillsbury EP, Ahrar K, Bessoud B, Hicks ME (2005). Splenic arterial interventions: anatomy, indications, technical considerations, and potential complications. Radiographics.

[CR28] Malhotra AK, Carter RF, Lebman DA, Carter DS, Riaz OJ, Aboutanos MB (2010). Preservation of splenic immunocompetence after splenic artery angioembolization for blunt splenic injury. J Trauma.

[CR29] McIntyre LK, Schiff M, Jurkovich GJ (2005). Failure of nonoperative management of splenic injuries: causes and consequences. Arch Surg.

[CR30] Mebius RE, Kraal G (2005). Structure and function of the spleen. Nat Rev Immunol.

[CR31] Moore EE, Cogbill TH, Jurkovich GJ, Shackford SR, Malangoni MA, Champion HR (1994). Organ injury scaling: spleen and liver (1994 revision). J Trauma.

[CR32] Nance FC, Nance ML (1995). Delayed presentation of splenic artery pseudoaneurysms following blunt abdominal trauma. J Trauma.

[CR33] Norotsky MC, Rogers FB, Shackford SR (1995). Delayed presentation of splenic artery pseudoaneurysms following blunt abdominal trauma: case reports. J Trauma.

[CR34] Okahara M, Mori H, Kiyosue H, Yamada Y, Sagara Y, Matsumoto S (2010). Arterial supply to the pancreas; variations and cross-sectional anatomy. Abdom Imaging.

[CR35] Olthof DC, Lammers AJ, van Leeuwen EM, Hoekstra JB, ten Berge IJ, Goslings JC (2014). Antibody response to a T-cell-independent antigen is preserved after splenic artery embolization for trauma. Clin Vaccine Immunol.

[CR36] Pirasteh A, Snyder LL, Lin R, Rosenblum D, Reed S, Sattar A (2012). Temporal assessment of splenic function in patients who have undergone percutaneous image-guided splenic artery embolization in the setting of trauma. J Vasc Interv Radiol.

[CR37] Requarth JA, D'Agostino RB, Miller PR (2011). Nonoperative management of adult blunt splenic injury with and without splenic artery embolotherapy: a meta-analysis. J Trauma.

[CR38] Rong J, Liu D, Liang M (2017). The impacts of different embolization techniques on splenic artery embolization for blunt splenic injury: a systematic review and meta-analysis. Mil Med Res.

[CR39] Saad WE (2012). Nonocclusive hepatic artery hypoperfusion syndrome (splenic steal syndrome) in liver transplant recipients. Semin Interv Radiol.

[CR40] Scatliff JH, Fisher ON, Guilford WB, McLendon WW (1975). The “starry night” splenic angiogram. Contrast material opacification of the malpighian body marginal sinus circulation in spleen trauma. Am J Roentgenol Radium Therapy, Nucl Med.

[CR41] Schnuriger B, Inaba K, Konstantinidis A, Lustenberger T, Chan LS, Demetriades D (2011). Outcomes of proximal versus distal splenic artery embolization after trauma: a systematic review and meta-analysis. J Trauma.

[CR42] Sclafani SJ, Weisberg A, Scalea TM, Phillips TF, Duncan AO (1991). Blunt splenic injuries: nonsurgical treatment with CT, arteriography, and transcatheter arterial embolization of the splenic artery. Radiology.

[CR43] Tsaroucha AK, Pitiakoudis MS, Chanos G, Chiotis AS, Argyropoulou PI, Prassopoulos P, Simopoulos CE (2005). U-stitching splenorraphy technique: experimental and clinical study. ANZ J Surg.

[CR44] Uranus S, Pfeifer J (2001). Nonoperative treatment of blunt splenic injury. World J Surg.

[CR45] Vaidya S, Tozer KR, Chen J (2008). An overview of embolic agents. Semin Interv Radiol.

[CR46] Warshaw AL (1988). Conservation of the spleen with distal pancreatectomy. Arch Surg.

[CR47] Widlus DM, Moeslein FM, Richard HM (2008). Evaluation of the Amplatzer vascular plug for proximal splenic artery embolization. J Vasc Interv Radiol.

[CR48] Zmora O, Kori Y, Samuels D, Kessler A, Schulman CI, Klausner JM (2009). Proximal splenic artery embolization in blunt splenic trauma. Eur J Trauma Emerg Surg.

